# Design and Functional Characterization of HIV-1 Envelope Protein-Coupled T Helper Liposomes

**DOI:** 10.3390/pharmaceutics14071385

**Published:** 2022-06-30

**Authors:** Dominik Damm, Ehsan Suleiman, Hannah Theobald, Jannik T. Wagner, Mirjam Batzoni, Bianca Ahlfeld (née Kohlhauser), Bernd Walkenfort, Jens-Christian Albrecht, Jidnyasa Ingale, Lifei Yang, Mike Hasenberg, Richard T. Wyatt, Karola Vorauer-Uhl, Klaus Überla, Vladimir Temchura

**Affiliations:** 1 Institute of Clinical and Molecular Virology, Friedrich-Alexander University Erlangen-Nürnberg, 91054 Erlangen, Germany; dominik.damm@uk-erlangen.de (D.D.); htheobal@uni-bonn.de (H.T.); jannik.wagner@uk-erlangen.de (J.T.W.); jens-christian.albrecht@uk-erlangen.de (J.-C.A.); klaus.ueberla@fau.de (K.Ü.); 2 Department of Biotechnology, University of Natural Resources and Life Sciences, 1190 Vienna, Austria; ehsansuleiman@gmx.at (E.S.); miri.batzoni@googlemail.com (M.B.); biancak1708@gmail.com (B.A.); karola.vorauer-uhl@boku.ac.at (K.V.-U.); 3 Electron Microscopy Unit (EMU), Imaging Center Essen (IMCES), Faculty of Medicine, University of Duisburg-Essen, 45147 Essen, Germany; bernd.walkenfort@uni-due.de (B.W.); mike.hasenberg@uni-due.de (M.H.); 4 Vaccine Business Unit, Takeda Pharmaceuticals, Cambridge, MA 02139, USA; jidnyasa.ingale@gmail.com; 5 Department of Immunology and Microbiology, The Scripps Research Institute, La Jolla, CA 92037, USA; lfyang@scripps.edu (L.Y.); wyatt@scripps.edu (R.T.W.)

**Keywords:** T helper liposomes, Env trimer, coupling mechanism, peptide encapsulation, intrastructural help, HIV, functionalization, vaccine, immunogold staining, GMP

## Abstract

Functionalization of experimental HIV-1 virus-like particle vaccines with heterologous T helper epitopes (T helper VLPs) can modulate the humoral immune response via intrastructural help (ISH). Current advances in the conjugation of native-like HIV-1 envelope trimers (Env) onto liposomes and encapsulation of peptide epitopes into these nanoparticles renders this GMP-scalable liposomal platform a feasible alternative to VLP-based vaccines. In this study, we designed and analyzed customizable Env-conjugated T helper liposomes. First, we passively encapsulated T helper peptides into a well-characterized liposome formulation displaying a dense array of Env trimers on the surface. We confirmed the closed pre-fusion state of the coupled Env trimers by immunogold staining with conformation-specific antibodies. These peptide-loaded Env-liposome conjugates efficiently activated Env-specific B cells, which further induced proliferation of CD4+ T cells by presentation of liposome-derived peptides on MHC-II molecules. The peptide encapsulation process was then quantitatively improved by an electrostatically driven approach using an overall anionic lipid formulation. We demonstrated that peptides delivered by liposomes were presented by DCs in secondary lymphoid organs after intramuscular immunization of mice. UFO (uncleaved prefusion optimized) Env trimers were covalently coupled to peptide-loaded anionic liposomes by His-tag/NTA(Ni) interactions and EDC/Sulfo-NHS crosslinking. EM imaging revealed a moderately dense array of well-folded Env trimers on the liposomal surface. The conformation was verified by liposomal surface FACS. Furthermore, anionic Env-coupled T helper liposomes effectively induced Env-specific B cell activation and proliferation in a comparable range to T helper VLPs. Taken together, we demonstrated that T helper VLPs can be substituted with customizable and GMP-scalable liposomal nanoparticles as a perspective for future preclinical and clinical HIV vaccine applications. The functional nanoparticle characterization assays shown in this study can be applied to other systems of synthetic nanoparticles delivering antigens derived from various pathogens.

## 1. Introduction

In recent years, the interest in HIV-1 nanoparticle vaccines has drastically increased. New platforms are being explored to improve immunization strategies for the induction of HIV-1 antibody responses [1]. Although both CD4+ T cells and B cells are involved in the humoral response to HIV-1 nanoparticle vaccines, their antigen recognition is based on two entirely different mechanisms. The efficient initiation of an adaptive humoral immune response against the HIV-1 surface glycoprotein (Env) requires Env-specific B cells to encounter non-degraded nanoparticles in the B cell areas of secondary lymphoid organs where the repetitive antigen display and particulate form together with the nano-size initiate primary B cell responses [2]. The initiated B cells must also obtain help from CD4+ T cells, because priming of Env-specific IgG antibody responses greatly depends on T cell help [3]. In contrast to B cells, antigen recognition and antigen-mediated help by CD4+ T cells are restricted to short linear epitopes, which might derive from different parts of the antigen. However, vaccine-induced T helper cells specific for epitopes of HIV-1 viral components may act as targets for infection and, thus, increase the viral transmission risk [4,5,6].

In general, pre-existing CD4+ T cells that are specific for internal viral components can be reactivated after immunization with virus-like particles (VLPs) or an attenuated virus and provide help for B cells specific for surface proteins of the virus to promote and modulate the humoral immune response [7,8]. Recently, this mechanism, termed intrastructural help (ISH), was applied to enhance and modulate HIV-specific humoral immune responses using experimental HIV-1 virus-like particle vaccines that incorporated heterologous (HIV-1 unrelated) peptide epitopes (T helper VLPs) [9,10]. Furthermore, recently obtained experimental evidence indicates that the formation of vaccine-induced HIV-specific T helper cells, suspected to increase the susceptibility for infection, could be impeded by heterologous ISH [11]. The main requirements for safe HIV-1 vaccines that exploit ISH are (i) a nanoparticulate structure that allows entry to the draining lymph nodes (LN), (ii) B cell receptor (BCR)-dependent uptake of the nanoparticles by surface antigen-specific B cells and (iii) the presentation of particle-derived, heterologous T helper epitopes on MHC-II molecules [11,12].

HIV-based lentiviral VLP vaccines, which were extensively used in preclinical ISH trials, are commonly produced in eukaryotic cell lines, thereby being wrapped into a lipid bilayer containing Env and producer cell-derived surface proteins [13]. Both the producer cell proteins and the HIV-based structural capsid components are unneeded for ISH-driven vaccination and may be detrimental for GMP production and clinical application [14].

On the contrary, synthetic nanoparticles usually have a custom-made design that can be optimized for GMP production. Lipid-based nanoparticles have become of recent interest in terms of vaccine design with the development of licensed vaccines against Influenza A virus (Inflexal^®^ V), Hepatitis A virus (Epaxal^®^) and SARS-CoV-2 (Comirnaty^®^, Spikevax^®^) [15,16,17]. Liposomes, being spherical vesicles consisting of a lipid bilayer with an aqueous cavity, have a close resemblance to enveloped VLPs. The additional establishment of soluble Env trimers, stabilized in the closed, prefusion conformation (SOSIP gp140 [18,19]), rendered it possible to produce liposomes that present a dense array of orthogonally coupled Env spikes on the surface. Env-coupled liposomes were superior over soluble Env trimers in the activation of Env-specific B cells in vitro and showed increased immunogenicity in vivo [20]. Furthermore, it was hypothesized that the site-directed coupling masks immunodominant, non-neutralizing epitopes at the base of the Env trimer [21]. Additionally, sequential immunization of rabbits and non-human primates with Env-coupled liposomes induced neutralizing humoral immune responses in both animal models [22,23].

Moreover, sophisticated protocols for efficient and reliable encapsulation of peptides and other pharmaceutics into liposomal formulations have recently been established. These methods include electrostatically driven encapsulation [24] and various microfluidic techniques [25,26]. 

In the current study, we generated T helper liposomes (lipid nanoparticles that display the B cell-targeting Env antigen on the surface and contain T helper cell epitopes in the core) by two different approaches. An array of in vitro methods was established and adapted to functionally characterize these nanoparticles in terms of Env trimer conformation, quantitative peptide encapsulation, activation of Env-specific B cells as well as the uptake and peptide presentation to T helper cells. 

## 2. Materials and Methods

### 2.1. Animals

Female wild-type C57BL/6NRj (Janvier, Le Genest-Saint-Isle, France) and T cell receptor-transgenic mice, specific for the OVA_323-339_ (OT2) peptide (OT2 mice [27]; in-house breeding; kindly provided by Dr. Diana Dudziak), and B cell receptor-transgenic mice specific for the HIV-1 surface glycoprotein Env (PGT121 [28] or b12 [29] mice; in-house breeding; kindly provided by Dr. Michel Nussenzweig and Dr. David Nemazee, respectively) of both sexes were used in this study.

### 2.2. Env Trimer Purification

JRFL Env trimers were produced and purified as previously described [19]. Additionally, we used the plasmid encoding for conSOSL.UFO.664 [30] to generate SUFO.664 trimers with a C-terminal His-tag (SUFO.664-His). To this end, the SUFO.664 open reading frame was cloned into a pcDNA 3.1+ backbone via NheI and XbaI restriction for an improved expression in HEK293F cells. Subsequently, a nucleotide sequence coding for a 1× G4S spacer and a 8x His-tag (GGCGGAGGAGGCTCCCACCATCATCACCACCATCACCAT) was inserted directly upstream of the stop codon. One liter of HEK293F cell culture, at a density of 1.0–1.2 × 10^6^ cells/mL, was transfected with 1 mg of SUFO.664-His DNA using linear PEI (Polysciences, Warrington, PA, USA) as transfection reagent. Cell supernatants were harvested 72 h after transfection and sterile-filtered. SUFO.664-His trimers were further purified by gravity-flow Galanthus Nivalis lectin (Vector Laboratories, Burlingame, CA, USA) affinity chromatography followed by size-exclusion chromatography using a HiPrep™ 26/60 Sephacryl^®^ S-300 HR column on a ÄKTA Purifier-10 system (both GE Healthcare, Chicago, IL, USA). Eluted protein fractions were analyzed by reducing SDS-PAGE, Western Blot, Native-PAGE and silver staining, as described previously [11]. Trimer fractions were pooled and concentrated using Amicon 10 kDa cutoff centrifuge filters (Merck Millipore, Billerica, MA, USA). Protein concentration was defined by (i) NanoDrop measurement, as previously described [11] and (ii) by ELISA. In short, a serial dilution of Env was captured by preblocked plates coated with anti-glycan antibody 2G12. After two further incubation steps with biotinylated PGT145 antibody and Streptavidine-POD conjugate (Roche, Penzberg, Germany; all antibodies from Polymun Scientific GmbH, Klosterneuburg, Austria), the signals were induced by adding H_2_O_2_-supplemented OPD substrate. The reactions were stopped by adding 25% H_2_SO_4_ and ODs were measured at 492 nm. Env concentrations were then calculated by comparison with the standard row signals.

### 2.3. Nanoparticle Preparation

#### 2.3.1. First-Generation Liposomes

First-generation Env-coupled liposomes were manufactured by thin-film hydration, as previously described [20], with slight modifications: After drying in a desiccator, the lipid film was hydrated with 1 mg/mL peptide in PBS for 3 h at 37 °C under constant shaking. Ovalbumine-derived peptide OT2 (OVA_323-339_; ISQAVHAAHAEINEAGR) was used to generate 1/~/JRFL/OT2. Hydration was performed with PBS alone to produce empty liposomes (1/~/JRFL//). Liposomal extrusion was performed on a heating block and the temperature of the extruder (hand-held mini-extrusion device, Avanti Polar Lipids, Alabaster, AL, USA) was maintained at 40 °C to avoid peptide loss. An amount of 2.2 mg of C-terminal His-tagged JRFL NFL trimers was added to 500 µL of the DGS-NTA(Ni)-containing liposomes for protein conjugation, incubating for 2 h at 37 °C. As a control, some peptide-encapsulating liposomes were not coupled with Env (uncoupled liposomes; 1/~//OT2). All liposomes were then cooled down to 4 °C and unbound Env, as well as free peptides, were subsequently removed by size-exclusion chromatography using a Superdex 100 column (GE Healthcare, Chicago, IL, USA). Nanoparticle analysis was performed as previously described [20,31]. 

#### 2.3.2. Uncoupled Anionic T Helper Liposomes

The generation of anionic T helper liposomes by thin-film hydration with a lipid composition of 45 mol% DSPC, 40 mol% cholesterol and 15 mol% DSPG was previously described in detail [24]. OT2 peptide solution was used for hydration of the lipid film. Subsequently, extrusion was performed at 55 °C to minimize peptide loss using a LIPEX™ Extruder (Northern Lipids Inc., Burnaby, BC, Canada) with Whatman^®^ Nucleopore track-etched membranes (5 cycles with 400 nm pore size, then 5–10 cycles with 200 nm or 100 nm pore sizes). Finally, the nanoparticle suspension was sterile-filtered (0.2 µm) and stored at 4 °C. Non-encapsulated peptides were removed by tangential flow filtration using a 100 kDa mPES MicroKros^®^ module (Repligen, Waltham, MA, USA) with a surface area of 20 cm^2^ [24].

#### 2.3.3. Second-Generation Anionic T Helper Liposomes

Double-functionalized, anionic T helper liposomes (OT2-loaded liposomes that contain both carboxyl- and NTA(Ni)-functionalized lipids) were prepared by thin-film hydration as described elsewhere [24]. Liposomes were composed of 77 mol% DOPC, 15 mol% DOPG, 4 mol% DSPE-PEG_14_-COOH and 4 mol% 18:1 DGS-NTA(Ni). Covalent conjugation of the His-tagged Env trimers was achieved by an EDC/Sulfo-NHS-based approach. A 40-fold molar excess of Sulfo-NHS and a 100-fold molar excess of EDC over the accessible carboxyl groups on the double-functionalized, peptide-loaded liposomes were used for activation. Activation was performed in 50 mM MBS pH 6.1 at a final concentration of 14.1 mM total lipid (282 µM accessible carboxyl-functionalized lipid). The reaction was then allowed to proceed for 15 min at 25 °C and 1400 rpm. Excess activation reagents were removed by gel filtration through NAP-5 columns (GE Healthcare UK Limited, Chalfont St Giles, UK). Liposomes were eluted with 10 mM PBS pH 7.5 w/150 mM NaCl. The activated liposomes were then further diluted and mixed with His-tagged Env trimers to give a final concentration of 1.7 mM total lipid (34 µM accessible NTA (Ni)-functionalized lipid) and 278 nM (100 µg/mL) His-tagged Env. This corresponds to a 40-fold molar excess of accessible NTA(Ni) over the His-tags of the trimeric HIV envelope protein. The conjugation reaction was then allowed to proceed for 4 h at 25 °C and 1400 rpm. After that, the reaction was quenched with a 10-fold molar excess of glycine over the accessible carboxyl groups on the liposomes. The Env-liposome conjugates were purified and concentrated by means of tangential flow filtration. Finally, they were sterile-filtered and stored at 2–8 °C and analyzed as previously described [24,32].

#### 2.3.4. Peptide Quantification via HPLC

The preparation and HPLC quantification of peptides extracted from T helper liposomes was described elsewhere [24]. In short, T helper liposomes were incubated in the presence of 80 mM sodium bicarbonate. An equal volume of n-butanol was added for lipid extraction in an Eppendorf ThermoMixer^®^ at 1400 rpm. After 15 min, the phases were separated by centrifugation at 3000× *g* for 10 min. The aqueous phase was collected and stored at −20 °C. Lipids and peptides were quantified using a 1200 series Agilent Technologies HPLC system (Agilent Technologies, Santa Clara, CA, USA), operated via ChemStation and on a Luna^®^ 5 µm C18 100 Å (150 × 4.6 mm) system (Phenomenex Inc., Torrance, CA, USA). All HPLC solvents were of LiChrosolv^®^ gradient grade (Merck KGaA, Darmstadt, Germany). Detailed calculations of the encapsulation efficiency and average number of peptides per liposome based on the HPLC results are described in the supplementary material of Suleiman et al. [24].

#### 2.3.5. T Helper VLPs

VLPs were produced as described previously [9,33]. Shortly, HEK293T cells were transiently transfected with plasmids encoding for membrane-bound conSOSL.UFO.750 [30] and a fusion protein consisting of HIV-GagPol and OT2 (Hgpsyn-OT2, [34]). At 64 h after transfection, cell supernatants were harvested and sterile-filtered. VLPs were pelleted by ultracentrifugation for 2.5 h at 28,000 rpm through a 35% sucrose cushion and resuspended in PBS. The concentration of Env and Gag in all VLP preparations was defined by a direct-coating ELISA using conS.gp140.CFI (CAVD Protein Production Facility, Duke Human Vaccine Institute, Durham, NC, USA) and recombinant p24 (Aalto Bio Reagents, Dublin, Ireland) as standard antigens, as well as 2G12 (Polymun Scientific, Klosterneuburg, Austria) and anti-p24 hybridoma antibody, respectively, for detection. Analytical methods to characterize VLPs were performed as described elsewhere [9,35]. 

### 2.4. Isolation of Murine Primary Cells

Primary cells isolated from mice were generally cultivated in RPMI 1640 (Gibco, Carlsbad, CA, USA) supplemented with 10% heat-inactivated FCS, penicillin-streptomycin, 10 μM HEPES, 4 μM L-glutamine and 50 μM β-mercaptoethanol (R10 medium) at 37 °C and 5% CO_2_. The cell density was determined using a Countess^TM^ Automated cell counter (Thermo Fisher, Waltham, MA, USA).

#### 2.4.1. B Cells

Splenic B cells were isolated from C57BL/6NRj (*wt*) mice or b12 [29] and PGT121 [28] B cell receptor-transgenic mice by magnetic cell separation (MACS; B Cell Isolation Kit (mouse), 130-090-862, Miltenyi Biotec, Bergisch-Gladbach, Germany) following the manufacturer’s guidelines. For preparation, the spleens were homogenized with a gentleMACS dissociator (Miltenyi Biotec, Bergisch-Gladbach, Germany) and filtered through a 70 µm nylon cell strainer (Fisher Scientific, Hampton, NH, USA). The cells were then incubated for 10 min at RT in ACK buffer (150 mM NH_4_Cl, 10 mM KHCO_3_, 0.1 mM EDTA) for erythrocyte lysis. The splenocytes were washed once in R10 medium before MACS separation was performed. 

#### 2.4.2. T Cells

Splenic CD4+ T cells were isolated from *wt* or OT2 T cell receptor-transgenic mice by MACS (CD4+ T Cell Isolation Kit (mouse), 130-104-454, Miltenyi Biotec, Bergisch-Gladbach, Germany) following the manufacturer’s guidelines. The splenocyte preparation was performed as described above for B cell isolation.

#### 2.4.3. Dendritic Cells (DCs)

Splenic DCs were isolated from *wt* mice by MACS (CD11c MicroBeads UltraPure (mouse), 130-125-835, Miltenyi Biotec, Bergisch-Gladbach, Germany) following the manufacturer’s guidelines. Before homogenization, tissue-degrading buffer (RPMI 1640 supplemented with 2 mg/mL collagenase and 0.1 mg/mL DNaseI) was injected into the spleens. The spleens were then incubated in tissue-degrading buffer for 45 min at 37 °C. The tissue was homogenized by filtering through a 70 µm nylon cell strainer. Erythrocytes were lysed as described above.

#### 2.4.4. Lymphocytes

Inguinal lymph nodes were isolated from *wt* mice and homogenized by using 70 µm nylon cell strainers. The lymphocytes were further used in different assays without ACK lysis.

### 2.5. Analysis of B Cell Activation and Proliferation

Splenic *wt*, b12 and PGT121 B cells were isolated by MACS as described above. Half of the B cells were then labeled with carboxyfluorescein succinimidyl ester (CFSE) to monitor proliferation using the Cell Trace™ CFSE kit (Thermo Fisher, Waltham, MA, USA) following the manufacturer’s protocol. Unlabeled B cells were used as proliferation controls and for activation assays. Amounts of 1.5 × 10^5^ CFSE+ cells for proliferation and 2 × 10^5^ unlabeled B cells for activation assays were seeded, per well, into U-bottom 96-well microplates (Greiner Bio-One, Kremsmünster, Austria). The B cells were incubated with liposomes at total Env concentrations of 1 µg/mL, 0.2 μg/mL and 0.04 μg/mL. Mock stimulation with PBS without bivalent cations (PBS/O) as well as lipopolysaccharide (LPS) (Sigma-Aldrich, Corp., St. Louis, MO, USA) stimulation were performed as negative and positive induction controls, respectively. After 24 h, the activation of the B cells was analyzed by immunostaining for B cell activation markers CD80, CD40, CD69 and CD62L as well as the B cell marker CD19 and a life/dead staining (anti-CD80-FITC (16-10A1), anti-CD62L-APC (MEL14), anti-CD40-PE (1L10), Fixable Viability Dye eFluor450 (all eBioscience™, Thermo Fisher, Waltham, MA, USA); anti-CD69-PE/Cy7 (H1.2F3) and anti-CD19-PerCP (6D5) (both BioLegend, San Diego, CA, USA)). The stained samples were measured using a benchtop BD™ LSR-II flow cytometer (BD Biosciences, Heidelberg, Germany). The CSFE-labeled B cells were evaluated for proliferation after 72 h by co-staining with Fixable Viability Dye eFluor450 and anti-CD45R/B220-APC (eBioscience™, Thermo Fisher, Waltham, MA, USA) and subsequent measurment by flow cytometry.

### 2.6. T Cell Proliferation by T Cell/Dendritic Cell Co-Culture

CD4+ T cells (*wt* and OT2-specific) and *wt* DCs were isolated and purified as described above. The T cells were additionally labeled with CFSE as described for B cells to monitor proliferation. In the following, 5 × 10^4^ CFSE-labeled T cells were seeded together with 2 × 10^5^ DCs into U-bottom 96-well microplates (Greiner Bio-One, Kremsmünster, Austria). The cells were incubated with either T helper liposomes or OT2 peptide at total bulk OT2 concentrations of 0.1 µM, 0.03 μM and 0.01 µM at 37 °C and 5% CO_2_. After 72 h, the cells were blocked with anti-mouse CD16/CD32 (93) and stained with both anti-mouse-CD4-APC (RM4-5) and Fixable Viability Dye eFluor™ 450 (all eBioscience™, Thermo Fisher, Waltham, MA, USA). Data were acquired using the BD™ LSR-II (BD Biosciences, Heidelberg, Germany) and analyzed with the FlowJo software (BD Biosciences, Franklin Lakes, NJ, USA). 

### 2.7. Ex Vivo DC Transfer

*Wt* mice were intramuscularly immunized with anionic OT2-liposomes or OT2 peptide (5 µg OT2 per dose) non-adjuvanted or adjuvanted with 10 µg poly-ICLC (Hiltonol^®^, Oncovir Inc., Washington, DC, USA) per mouse. The mice were sacrificed 21 h later and both spleens and inguinal lymph nodes were isolated. Lymphocytes, as well as purified 6 × 10^5^ DCs from immunized mice, were co-cultured with 2 × 10^5^ CFSE-labeled, OT2-specific T cells for 72 h. The cells were then stained with Fixable Viability Dye and a CD4 antibody (Thermo Fisher, Waltham, MA, USA) and T cell proliferation was analyzed by flow cytometry (LSR-II, BD, Franklin Lakes, NJ, USA) based on CFSE signal distributions.

### 2.8. In Vitro Intrastructural Help

Primary *wt* or OT2-specific T cells, as well as b12 B cells, were isolated by magnetic cell separation as described above. Co-cultures of 1 × 10^5^ Env-specific B cells with 1 × 10^5^ CFSE-labeled T cells were prepared and incubated with liposomal formulations at a bulk Env concentration of 2 µg/mL. After 72 h, the cells were Fc-blocked with anti-mouse CD16/CD32 (93) and stained with anti-mouse-CD4-APC (BD Pharmingen, Franklin Lakes, NJ, USA) and Fixable Viability Dye eFluor™ 450. The CFSE distribution within CD4+ cells was measured using the BD™ LSR-II flow cytometer.

### 2.9. Antibody Staining of Liposomes

#### 2.9.1. Immunogold EM Imaging of T Helper Liposomes

For all TEM analyses, T helper liposome suspensions were diluted 1:50 in PBS (137 mM NaCl, 2.7 mM KCl, 10 mM Na_2_HPO_4_, 1.8 mM NaH_2_PO_4_, pH 7.4) and 7 µL drops of these dilutions were incubated for 10 min on Formvar/carbon supported 400 mesh nickel grids (#S162N9, Plano GmbH, Wetzlar, Germany; “drop-on-grid” method). If not stated differently, these, and all subsequent steps, were performed at room temperature. For pure negative staining, the liposome suspension was then carefully removed from the grid by use of filter paper, followed by three 5 min washing steps with deionized H_2_O (“grid-on-drop” method with 30 µL drops on Parafilm^®^). Excess water was then removed from the grid using filter paper. The grids were incubated for 1 min on a 10 µL drop of an aqueous 1.5% phosphotungstic acid (PTA) solution. After incubation, the PTA solution was immediately removed with a piece of filter paper, grids were air-dried for a couple of minutes and samples were examined with a JEOL JEM-1400Plus, operating at 120 kV and equipped with a 4096 × 4096 pixels CMOS camera (TemCam-F416, TVIPS, Gauting, Germany). 

For immunogold staining, a panel of monoclonal anti-Env antibodies (2G12, Polymun Scientific GmbH, Klosterneuburg, Austria; 17b, PGT145, NIH AIDS Reagent Program, Bethesda, MD, USA) was coupled to 10 nm gold nanoparticles using a commercial conjugation kit (#ab201808, Abcam, Cambridge, UK) following the manufacturer’s guidelines. After liposome adherence to the grid by the “drop-on-grid” protocol (see above), three 5 min washing steps with PBS were conducted (“grid-on-drop” method with 30 µL drops). After blocking with PBS+-buffer (137 mM NaCl, 2.7 mM KCl, 10 mM Na_2_HPO_4_, 1.8 mM KH_2_PO_4_, 5% *w*/*v* BSA, 1.5% *w*/*v* glycine; pH 7.4) for 10 min, excess PBS+ was removed using a piece of filter paper. The grids were placed on single drops of diluted antibody solutions (75 ng/µL stocks diluted 1:25 in PBS+ buffer) overnight at 4 °C. Next day, negative staining and image acquisition was performed as stated above, starting with the three deionized water washing steps.

#### 2.9.2. Liposomal Surface FACS

Liposomes were diluted in PBS/O supplemented with 1% BSA and 1 mM EDTA. They were further stained with monoclonal Env antibodies (2G12, PG9, Polymun Scientific GmbH, Klosterneuburg, Austria; 17b, PGT121, PGT145, 447-52D, NIH AIDS Reagent Program, Bethesda, MD, USA) and anti-hCMV gB antibody 27-287 (kindly provided by Prof. Michael Mach, Virologisches Institut, Universitätsklinikum Erlangen, Germany). Here, the amount of antibody was in 10-fold excess to the amount of Env on liposomes and the reaction volume did not exceed 30 µL. After 30 min incubation on ice, the volume was increased to 100 µL by adding anti-human-IgG-AlexaFluor^®^488 in PBS/O (BioLegend, San Diego, CA, USA), thereby making the final secondary antibody dilution 200-fold. The samples were again incubated for 30 min on ice. Finally, 200 µL buffer was added and antibody binding was evaluated by flow cytometry using a BD™ LSR-II (Becton Dickinson, Franklin Lakes, NJ, USA). 

### 2.10. Statistical Analysis

Figures and statistics were created using the GraphPad Prism 7 software (GraphPad Software Inc., San Diego, CA, USA).

## 3. Results and Discussion

T helper nanoparticles display the antigen of interest on the surface and encapsulate heterologous T helper cell epitopes in the core. In the context of the HIV-1 humoral immune response, intrastructural help (ISH) requires the efficient B cell receptor-dependent uptake of such T helper nanoparticles by Env-specific B cells. The nanoparticles are then processed within the cells’ lysosomal compartments and particle-derived peptides are presented on MHC-II surface molecules. This enables heterologous (non-Env-specific) T helper cells induced by previous immunizations to recognize their target epitope and to promote Env-specific B cell responses. This process has been thoroughly evaluated with lentiviral T helper VLPs in the past [10,35,36]. More recent data have indicated that synthetic, inorganic T helper nanoparticles are also processed appropriately and can be harnessed to induce in vivo ISH effects [11,36]. 

In the following, two ways of generating T helper liposomes are presented. The nanoparticles were compared in terms of functionality by several assays addressing the surface conjugation of Env trimers and the quantitative encapsulation as well as the MHC-II presentation of heterologous peptides. A description of all nanoparticles characterized in this study is given in Table 1. 

### 3.1. First-Generation T Helper Liposomes Produced by Passive Peptide Entrapment

As an initial attempt, we performed passive peptide entrapment in well-characterized liposomes displaying a dense array of stabilized JRFL NFL Env trimers coupled by a conformation-preserving mechanism [19,20]. T helper peptides were added in the lipid film hydration step during nanoparticle production, resulting in the first generation of T helper liposomes (1-Gen liposomes; 1/~/JRFL/OT2; Table 1).

**Figure 1:**
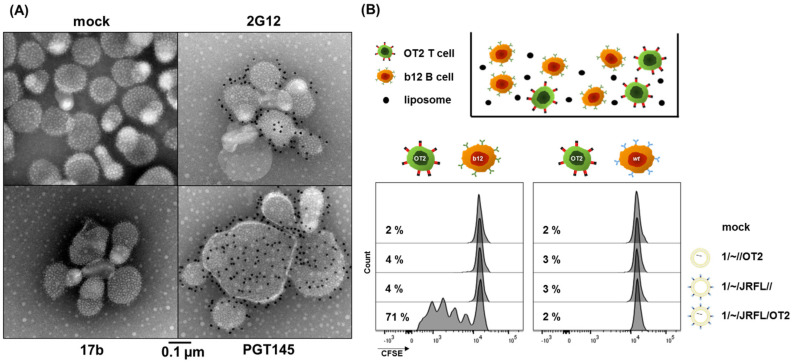
In vitro characterization of first-generation liposomes. (**A**) Immunogold staining of T helper liposomes. The conformation of the Env trimers on the surface of first-generation T helper liposomes was evaluated by negative stain TEM of liposomal samples treated with gold-coupled antibodies 17b, 2G12 and PGT145. The unstained liposomes appear as spherical particles with a dense array of Env trimers on the surface (upper left). Black dots on the EM images indicate binding of the respective antibodies to liposome-coupled Env. (**B**) In vitro intrastructural help. Co-cultures of 1 × 10^5^ b12 or wild-type (wt) B cells and 1 × 10^5^ CFSE-labeled OT2 T cells were incubated in the presence of different 1-Gen liposomal formulations (2 µg/mL bulk Env). After 72 h, T cell proliferation was analyzed by flow cytometry based on the CFSE dispersion.

To evaluate the Env conformation on T helper nanoparticles, we established immunogold staining of Env-coupled liposomes. The CD4-induced (CD4i) antibody 17b, as well as the glycan-specific antibody 2G12, and the apex-directed antibody PGT145, were coupled with gold beads (10 nm in diameter). First-generation liposomes were incubated with these gold-coupled antibodies and analyzed by transmission electron microscopy (Figure 1A). Antibody binding, represented by black dots on the images, was detected for both 2G12 and PGT145, but not for 17b. This clearly indicates that the closed prefusion state of the trimers was preserved on the liposomes. In previous studies, the correct Env conformation was commonly confirmed by propeller-like structures visualized with EM imaging [37,38]. Thus, immunogold staining of Env-coupled nanoparticles with conformation-specific antibodies might serve as a relevant extension of this method. 

It was previously shown that Env-coupled liposomes induce a significantly stronger activation of Env-specific b12 B cells than soluble trimers [20]. We compared 1-Gen T helper liposomes (1/~/JRFL/OT2) with Env-coupled liposomes (1/~/JRFL//) and uncoupled control liposomes (1/~//OT2) in an in vitro B cell activation assay (Figure S1). LPS served as a positive control for overall polyclonal B cell activation capacities. Compared to uncoupled 1/~//OT2 liposomes, both 1/~/JRFL/OT2 and 1/~/JRFL// induced significant upregulation of the activation marker CD69 and co-stimulatory molecules (CD40 and CD80), as well as significant downregulation of CD62 ligand (indicating BCR crosslinking) in a comparable scale. In addition, B cell viability was equal with both liposomal formulations. As expected, uncoupled liposomes (1/~//OT2) did not induce any activation of b12 B cells (no significance compared to mock control). These data confirm that passive peptide inclusion into 1/~/JRFL// has no influence on the antigen-mediated liposomal effects on cognate B cells. 

The presentation of peptides encapsulated into 1-Gen liposomes on MHC-II molecules was evaluated with an in vitro intrastructural help simulation. b12 B cells and CFSE-labeled OT2-specific CD4+ T cells were incubated for 72 h in the presence of different liposomal formulations (Figure 1B). T cell proliferation was only detectable in co-cultures incubated with 1/~/JRFL/OT2 T helper liposomes, but not in those with uncoupled 1/~//OT2 and empty 1/~/JRFL//, or when B cells isolated from wild-type mice were used. This clearly demonstrated that T helper liposomes were taken up in a BCR-dependent mechanism and that encapsulated peptides were presented on the B cell surface as prerequisites for T cell/B cell interaction. 

Despite the observed T cell proliferation in the functional in vitro assay (Figure 1B), HPLC analysis of the 1-Gen liposomes revealed that the peptide fraction was below the detection limit (data not shown). Although the precise number of epitope molecules required for efficient intrastructural help in vivo remains to be determined [9], we can estimate the number of epitopes in nanoparticles applied in previously published in vivo ISH experiments. Since T helper epitopes of VLPs were recombinantly encoded in the structural proteins of the particle, each T helper VLP contained a fixed number of epitopes, starting from 2 × 10^3^ epitopes per particle (depending on the T helper VLP design) [9,10]. Inorganic T helper calcium phosphate nanoparticles, recently applied for Tetanus-mediated ISH, contained 3.2 × 10^4^ Tetanus epitopes per particle [11].

However, the conventional, purely passive peptide entrapment (e.g., thin-film hydration of phospholipids in the presence of an aqueous suspension of peptides) leads to low encapsulation efficiencies, as the process proceeds in an uncontrolled manner [14] and is considered to be largely determined by the entrapped volume [39]. As a consequence, for the next generation of T helper liposomes, we decided to increase both the controllability of peptide encapsulation and the quantitative amount of encapsulated peptides. 

### 3.2. Functional Characterization of Uncoupled, Anionic Liposomes with Improved Peptide Encapsulation

In order to optimize the quantitative amount of peptides within liposomes, we changed the liposomal composition to apply a previously established and thoroughly characterized electrostatically driven approach with an encapsulation efficiency ~48% [24]. Using this method, we generated anionic liposomes which encapsulated ~10^3^ OT2 peptide molecules per particle (uncoupled anionic T helper liposomes; 2/-//OT2). The nanoparticle production was performed under optimized conditions in terms of pH, ionic strength and molar peptide : lipid ratio. The average number of peptides per liposome was calculated on the basis of the particle size distribution, the lipid concentration and the peptide concentration of the purified liposomes. The liposomes used for the functional assays in this study had a Z-Average of 122.7 ± 5.6 nm and an average polydispersity index (PdI) of 0.05 ± 0.01, as determined elsewhere [24]. A mean of 949 (~10^3^) OT2 peptides per liposome was calculated.

**Figure 2:**
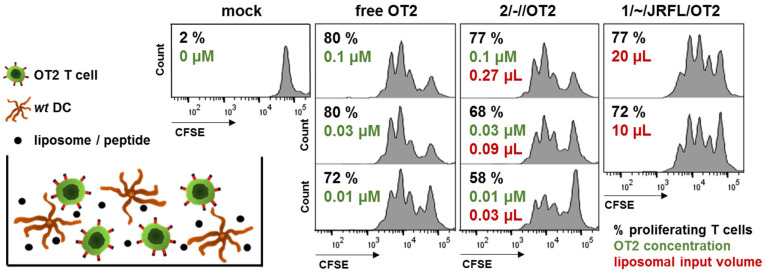
Functional comparison of peptide encapsulation in 2/-//OT2 and 1/~/JRFL/OT2. Co-cultures of 2 × 10^5^ wild-type dendritic cells (*wt* DCs) and 5 × 10^4^ CFSE-labeled OT2-specific T cells (OT2 T cell) were incubated in the presence of liposomal formulations (2/-//OT2 and 1/~/JRFL/OT2) or free OT2 peptide for 72 h. OT2 T cell proliferation was evaluated by flow cytometry based on the CFSE distribution in offspring cells. Proliferation in % (black), bulk OT2 concentration (green) and input volume of liposomal stock solution (red) are given for each culture well. The experiment was performed three times with comparable results.

As a next step, we compared 2/-//OT2 liposomes with previously produced 1/~/JRFL/OT2 nanoparticles in an in vitro assay, which was based on the unspecific uptake of the liposomes by primary DCs and the presentation of encapsulated peptides to cognate T cells on MHC-II molecules (Figure 2). DC/T cell co-cultures were incubated with either 2/-//OT2 or free OT2 peptide, which can directly bind to MHC-II from the supernatant, in a dilution series normalized to the bulk concentration of OT2. Because the amount of OT2 peptide present in 1-Gen liposomes was below HPLC detection limit, we titrated input volumes of the liposomal stock solutions in the co-cultures. A 74-fold to 111-fold volume of 1-Gen liposomes was necessary to induce the same range of OT2-specific T cell proliferation compared to 2/-//OT2 liposomes (20 µL vs. 0.27 µL; 10 µL vs. 0.09 µL). The titration series of liposomal stocks helped to identify the critical amount of lipids that caused cell death. Measurable levels of cytotoxicity started with a lipid concentration ≥ 240 µM (data not shown).

**Figure 3:**
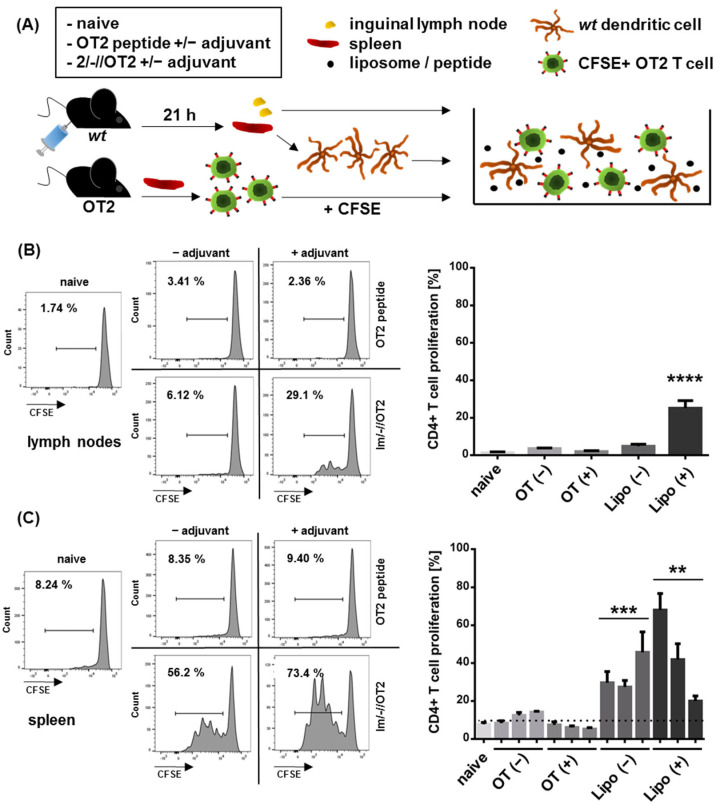
Ex vivo DC transfer. (**A**) Experimental procedure. C57bl/6 mice (n = 3) were intramuscularly vaccinated with OT2 peptide or anionic, uncoupled T helper liposomes encapsulating 10^3^ OT2 molecules (2/-//OT2) alone (OT(-), Lipo(-)) or adjuvanted with poly-ICLC (OT(+), Lipo(+)). The total amount of OT2 peptide per dose was 5 µg. After 21 h, the spleens and inguinal lymph nodes were isolated. Splenic DCs from single mice (6 × 10^5^), as well as pooled lymphocytes from one donor group, were then co-cultured with CFSE-labeled, OT2-specific T cells (2 × 10^5^) isolated from transgenic mice, respectively. T cell proliferation was measured three days later by flow cytometry. Shown are the representative histograms (left panel) and columns displaying CD4+ T cell proliferation (right panel) induced by DCs from pooled lymphocyte groups (**B**) and from spleens of single mice (**C**). Error bars indicate the standard deviations of the proliferation rates of OT2 T cells from two different mice that were incubated with APCs from one donor mouse. (**B**) **** *p* < 0.0001; ordinary one-way ANOVA with Dunnett’s multiple comparisons test. (**C**) ** *p* < 0.005; *** *p* < 0.001; unpaired two-tailed t test of liposome and peptide groups (means of shown individual mice) with matching adjuvantation.

We further addressed the in vivo stability of 2/-//OT2 and the presentation of encapsulated peptides in secondary lymphoid organs. To this end, an ex vivo adoptive DC transfer was performed. Wild-type C57bl/6 mice were intramuscularly immunized in both hind legs with either free OT2 peptide or 2/-//OT2 liposomes, adjuvanted or nonadjuvanted with TLR-3 ligand poly-ICLC [40]. On the following day, we isolated and co-cultured inguinal lymphocytes or purified splenic DCs from these mice with OT2-specific CD4+ T cells (Figure 3A). T cell proliferation was induced by both inguinal lymphocytes and splenic DCs, but only in mice that received 2/-//OT2 (Figure 3B,C). The proliferation was stronger in co-cultures with purified splenic DCs compared to co-cultures with total lymph node cell suspension. The effect might be due to the availability of APCs loaded with peptides in the assay, because the T:DC ratio plays a crucial role in the CD4 T cell activation in vitro [41]. With the purification of splenic DCs, we could unify the T:DC ratio for all co-cultures and additionally demonstrate that—after an intramuscular injection—splenic DCs might efficiently present peptides from 2/-//OT2 T helper liposomes to cognate CD4 T cells, even in the absence of an adjuvant. Due to limited total cell numbers from inguinal LNs, we performed co-cultures with whole-cell suspensions containing different amounts of APCs. In this setting, we were able to demonstrate the T cell proliferation for the adjuvanted liposomes, but not for the non-adjuvanted group. Importantly, although free OT2 peptides could directly bind to the MHC-II molecules on DCs and induce a potent OT2 T cell proliferation in vitro (Figure 2), this was not sufficiently effective in vivo (Figure 3).

Taken together, in comparison to the passive peptide entrapment, the electrostatically driven encapsulation efficiency was confirmed in both the analytical and functional approaches. The produced anionic liposomes encapsulated an average of 1 × 10^3^ OT2 epitopes per particle and, thus, were in the same range as the conventional T helper VLPs. The ex vivo adoptive transfer provided evidence that (i) charged T helper liposomes are stable in vivo and (ii) encapsulated T helper peptides are presented by APCs in secondary lymphoid organs after intramuscular immunization. This in vivo MHC-II presentation of liposome-derived peptides is a crucial requirement for intrastructural help.

### 3.3. Production of Second-Generation T Helper Liposomes by Covalent Coupling of Native-Like Env Trimers

The primary strategy for the coupling of stabilized JRFL NFL Env trimers to 1-Gen T helper liposomes was using a non-covalent interaction of His-tagged Env trimers and Ni-NTA lipids. This mechanism preserves Env trimer conformation (Figure 1) and provides conjugation stability in the buffer [19]. However, in the presence of serum, some degree of dissociation was reported [31]. Based on the thorough optimization and evaluation of 2/-//OT2 liposomes, we considered a tag-based mechanism to covalently couple state-of-the-art native-like Env trimers to the nanoparticle surface. 

**Figure 4:**
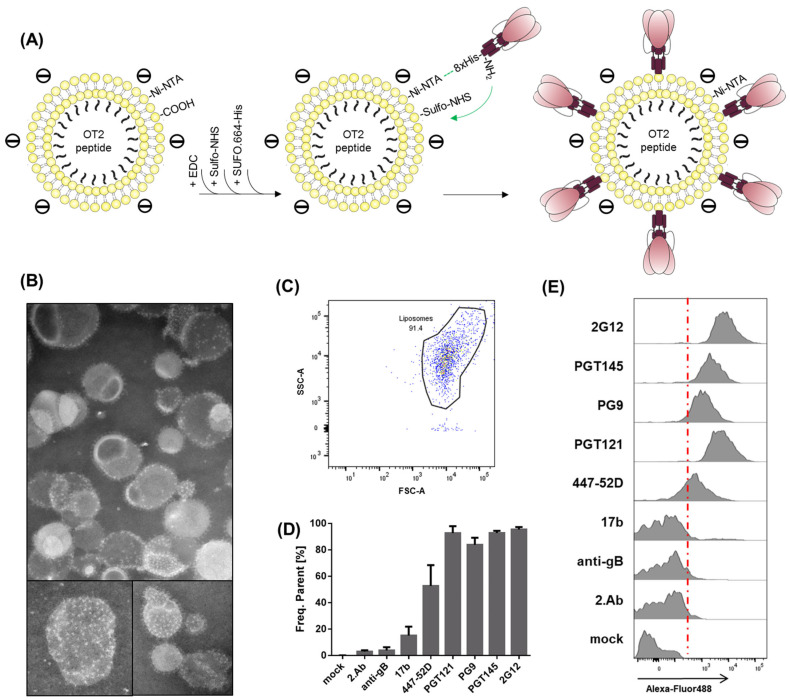
Production and characterization of second-generation liposomes. (**A**) Coupling mechanism. Double-functionalized 2/-//OT2 liposomes were produced by thin-film hydration including both DGS-NTA(Ni) and DSPE-PEG_14_-COOH in the lipid formulation. The carboxyl groups were activated by EDC/Sulfo-NHS. ConSOSL.UFO.664 trimers with a C-terminal 8x His-tag (SUFO.664-His) interacted with the Ni-NTA residues on the liposomal surface. The Sulfo-NHS esters then formed covalent bonds with primary amines of Env, thus coupling the trimers to the liposomes. (**B**) TEM imaging of second-generation liposomes. Coupled SUFO.664-His trimers appear as propeller-like structures on the liposomal surface. (**C**–**E**) Liposomal surface FACS staining. Second-generation liposomes were stained with a panel of monoclonal Env antibodies (2G12, PGT145, PG9, PGT121, 447-52D, 17b) and anti-human-AlexaFluor^®^488 secondary antibody. Controls were treated with a monoclonal anti-CMV glycoprotein B isotype antibody (anti-gB), secondary antibody alone (2.Ab) or with buffer only (mock). The liposome population was gated on a logarithmic FSC/SSC and a cutoff was set for fluorescent particles (red line). (**C**) Nanoparticles in FSC/SSC dot plot. (**D**) The columns represent the mean percentage + SD of antibody-bound liposomes from three independent experiments. (**E**) The binding histograms of the different antibodies.

We introduced an 8 × His-tag at the C-Terminus of the consensus S stabilized trimer conSOSL.UFO.664 (SUFO.664-His). The protein was expressed in 293F cells and purified by affinity chromatography with agarose-bound *Galanthus nivalis* lectin followed by size-exclusion chromatography. All protein elution fractions were analyzed by NativePAGE with subsequent silver stain and verified by Western Blot (Figure S2). Trimer-containing fractions were pooled and concentrated. Both DGS-NTA(Ni) and DSPE-PEG_14_-COOH were included in the lipid formulation to produce double-functionalized liposomes. SUFO.664-His trimers were brought into close proximity with the liposomal surface by His-tag/Ni-NTA interactions (Figure 4A). The carboxyl groups on the surface were used for EDC/Sulfo-NHS activation, generating amine-reactive esters. We hypothesized that these reactive groups preferentially formed covalent bonds with primary amines located in the gp41 ectodomains of the Env trimers, leading to the formation of second-generation (2-Gen), negatively charged T helper liposomes (2/-/SUFO/OT2). In general, the 2-Gen liposomes used in this study had a Z-average size of 124.7 nm, a mean polydispersity index (PdI) of 0.04, a mean zeta potential of −60.5 mV and encapsulated approximately 10^3^ OT2 peptide molecules. TEM imaging revealed that the 2/-/SUFO/OT2 liposomes presented a moderately dense array of well-ordered Env trimers on the surface. The coupling efficiency and uniformity varied among particles (Figure 4B). 

We addressed both the antigenicity as well as the conformation of coupled SUFO.664-His trimers by nanoparticle FACS analysis. 2/-/SUFO/OT2 liposomes were stained with a panel of monoclonal Env antibodies and matching AlexaFluor^®^488-positive secondary antibodies. The liposomes appeared as a population on a logarithmic FSC/SSC (Figure 4C). We evaluated the binding of the antibodies to the liposomes based on the percentage of AlexaFluor488-positive particles (Figure 4D) and fluorescence intensity (Figure 4E). This experimental approach was previously published for inorganic calcium phosphate nanoparticles orthogonally coupled with native-like Env trimers [42]. The best binding was observed with PGT121 and 2G12, both targeting heavily glycosylated sites on the protein surface and able to bind one trimer in a 3:1 ratio. More importantly, 2-Gen liposomes were strongly recognized by the trimer-specific apex antibodies PG9 and PGT145. This provides evidence that the coupling mechanism preserved the closed trimer conformation. The mean fluorescence intensities of liposomes incubated with 2G12 and PGT121, compared to those incubated with PG9 and PGT145, mirrored the antibody binding ratios to Env (3:1 vs. 1:1) [30,43,44]. However, we also observed a weak binding of the V3-loop antibody 447-52D, indicating that epitopes associated with an open or dissociated conformation of SUFO.664 might be present in a low degree. On the contrary, no binding was detected with the open-conformation CD4i antibody 17b. The human monoclonal anti-CMV gB antibody 27-287 [45] was used as an isotype control. 

**Figure 5:**
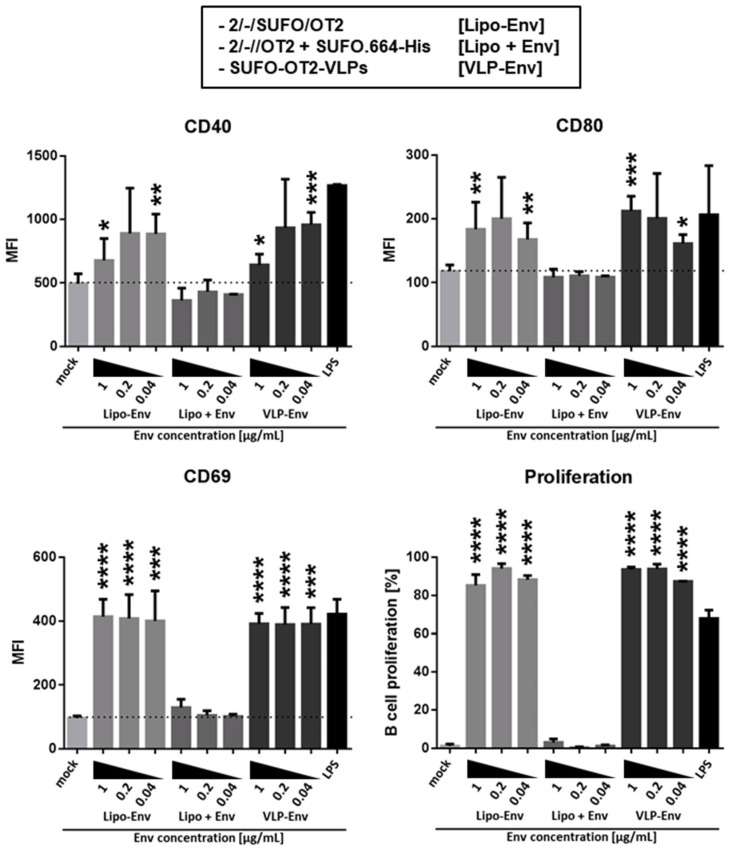
Functional evaluation of second-generation T helper liposomes. In vitro B cell activation assay with second-generation liposomes. Splenic PGT121 B cells—optionally labeled with a CFSE proliferation marker—were incubated with 2-Gen 2/-/SUFO/OT2 liposomes (Lipo-Env), lentiviral T helper SUFO-OT2-VLPs (VLP-Env) or a mixture of uncoupled 2/-//OT2 and soluble SUFO.664-His trimers (Lipo + Env) with matching bulk Env (1–0.04 µg/mL) and lipid concentrations. An amount of 2 × 10^5^ CFSE- B cells were seeded for activation assays and 1.5 × 10^5^ CFSE+ B cells were seeded for proliferation assays. As a positive control, B cells were incubated in the presence of 1 µg/mL LPS. Activation was defined by upregulation of markers CD40, CD80 and CD69. The mean values of the median fluorescence intensities (MFI) of three independent experiments are represented by the columns ± SD. * *p* < 0.05; ** *p* < 0.005; *** *p* < 0.001; **** *p* < 0.0001; ordinary one-way ANOVA and Tukey’s multiple comparisons test were conducted between groups with matching bulk Env concentrations; asterisks indicate significant differences of Lipo-Env and/or VLP-Env compared to Lipo + Env.

Based on the apparently strong PGT121 interaction, we tested the 2-Gen liposomes in an in vitro activation and proliferation assay with Env-specific B cells, which had been purified from the spleens of PGT121 B cell receptor-transgenic mice (kindly provided by Dr. Michel Nussenzweig, Rockefeller University, New York City, NY, USA). The experimental setup was similar to the one depicted in Figure S1. The T helper VLPs (SUFO-OT2-VLPs) were used as a positive control for the BCR-specific B cell activation; LPS served as a positive control for the overall polyclonal B cell activation. In order to test dose-dependency, we incubated the PGT121 B cells with a dilution series of nanoparticles normalized to the bulk concentration of Env in the assay (1–0.04 µg/mL; Figure 5). 2/-/SUFO/OT2 liposomes induced B cell activation and proliferation in a comparable range as the SUFO-OT2-VLPs, as seen by the upregulation of the B cell activation marker CD69, and the costimulatory markers CD40 and CD80. As a negative control, we incubated the PGT121 B cells in the presence of uncoupled 2/-//OT2 liposomes and soluble SUFO.664-His trimers (Lipo + Env) with matching dilutions of bulk peptide and Env concentrations. Both the T helper VLPs and liposomes induced significant B cell activation and proliferation compared to this control group. However, there were no significant differences between the two nanoparticle systems, stressing the hypothesis that VLPs can be replaced by our synthetic nanoparticles in future applications. We did not observe any levels of B cell activation and proliferation with the Lipo + Env control setup. These data indicate that (i) 2/-/SUFO/OT2 liposomes were functional in terms of B cell recognition and BCR-dependent uptake and (ii) there was no unspecific coupling of SUFO.664-His with double-functionalized 2/-//OT2 particles. Overall, the newly designed and functionally characterized 2/-/SUFO/OT2 liposomes meet the initial requirements for ISH nanoparticle vaccines.

## 4. Conclusions

T helper nanoparticles are defined by displaying the antigen of interest on the surface and encapsulating heterologous T helper epitopes in order to modulate humoral immune responses by intrastructural help. Lentiviral T helper VLPs were predominantly used for preclinical ISH in vivo experiments. However, the GMP scalability and clinical application of these nanoparticles is limited [14]. Therefore, we focused on the generation of synthetic T helper nanoparticles, which were produced by rational design, using sophisticated production protocols. These procedures certify scalability, as well as the high purity of the particles (i.e., no foreign antigens and no immunogenic buffer contaminants) and enable thorough functional in vitro analyses. We established and adapted an array of assays to characterize the T helper liposomes that were used in this study. The quantity, conformation and recognition of the surface-coupled native-like Env trimers were evaluated by immunogold TEM imaging, liposomal surface FACS and B cell activation/proliferation assays. The encapsulation efficiency and APC presentation of internal peptides were analyzed by T cell/DC co-cultures and ex vivo adoptive DC transfer. As a result, by the combination of a well-characterized electrostatically driven encapsulation approach with a yet unpublished covalent coupling strategy based on His-tag/Ni-NTA interactions and EDC/Sulfo-NHS crosslinking, we designed, produced and characterized second-generation T helper liposomes. These charged liposomes displayed a moderately dense array of well-ordered Env trimers on the surface and encapsulated an average of 1 × 10^3^ heterologous epitopes per particle. The liposomes (i) efficiently provided the presentation of particle-derived, heterologous T helper epitopes on the MHC-II molecules both in vitro and in vivo and (ii) demonstrated a B cell receptor-dependent activation of Env-specific B cells, comparable with T helper VLPs. Thus, these liposomes meet the initial requirements for ISH nanoparticle vaccines and will be used for future immunization experiments. Furthermore, the functional assays in this study can be adapted to future preparations of T helper nanoparticles, or functionalized nanoparticles in general, not only in the context of HIV vaccine research, but also based on antigens from different pathogens.

## Figures and Tables

**Table 1:** Overview of different liposomal preparations used in this study. Schematic pictures of the liposomes and VLPs (left panel), particle codes (center) and production description (right). Liposomal codes: [Gen]/[Charge]/[Env]/[Peptide].

First-generation T helper liposomes		
** 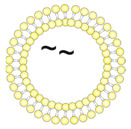 **	*1/~//OT2*	Uncoupled liposomes with zeta potential in a neutral range. OT2 peptides (black) were encapsulated by passive inclusion during hydration of the lipid film.
** 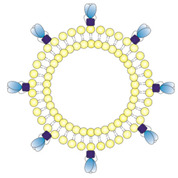 **	*1/~/JRFL//*	Empty liposomes with zeta potential in a neutral range. JRFL NFL trimers (blue) were coupled to the liposomal surface by interaction of C-terminal His-tags with Ni-NTA lipids.
** 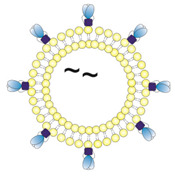 **	*1/~/JRFL/OT2*	T helper liposomes with zeta potential in a neutral range. OT2 peptides (black) were encapsulated by passive inclusion during hydration of the lipid film. JRFL NFL trimers (blue) were coupled to the liposomal surface by interaction of C-terminal His-tags with Ni-NTA lipids.
**Second-generation T helper liposomes**		
** 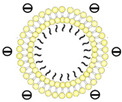 **	*2/-//OT2*	Uncoupled liposomes with an anionic zeta potential. OT2 peptides (black) were quantitatively encapsulated by an electrostatically driven approach.
** 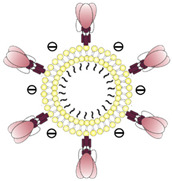 **	*2/-/SUFO/OT2*	T helper liposomes with an anionic zeta potential. OT2 peptides (black) were encapsulated by an electrostatically driven approach. conSOSL.UFO.664 trimers (red) were covalently coupled to the liposomal surface by His-tag / Ni-NTA interaction followed by EDC / Sulfo-NHS crosslinking.
**Lentiviral T helper VLPs**		
** 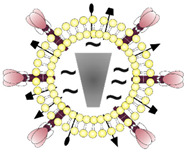 **	*SUFO-OT2-VLP*	T helper VLPs that display conSOSL.UFO.750 trimers (SUFO.750, red) and 293T producer cell-derived proteins (black) on the surface and encapsulate both HIV-1 capsid proteins (grey) and OT2 peptides (black).

## Data Availability

For any additional information, please contact the corresponding author.

## Supplementary Materials

The following supporting information can be downloaded at: https://www.mdpi.com/article/10.3390/pharmaceutics14071385/s1, Figure S1: In vitro B cell activation induced by first-generation liposomes; Figure S2: Size-exclusion (SEC) purification of SUFO.664-His.
