# Supplementary material for: Design and Functional Characterization of HIV-1 Envelope Protein-Coupled T Helper Liposomes
Source: Pharmaceutics. 2022 Jun 30;14(7):1385. doi: 10.3390/pharmaceutics14071385 (PMC9318220; doi:10.3390/pharmaceutics14071385)
Supplement: Supplementary file 1 [file pharmaceutics-14-01385-s001.zip › pharmaceutics-1774315-supplementary.pdf]

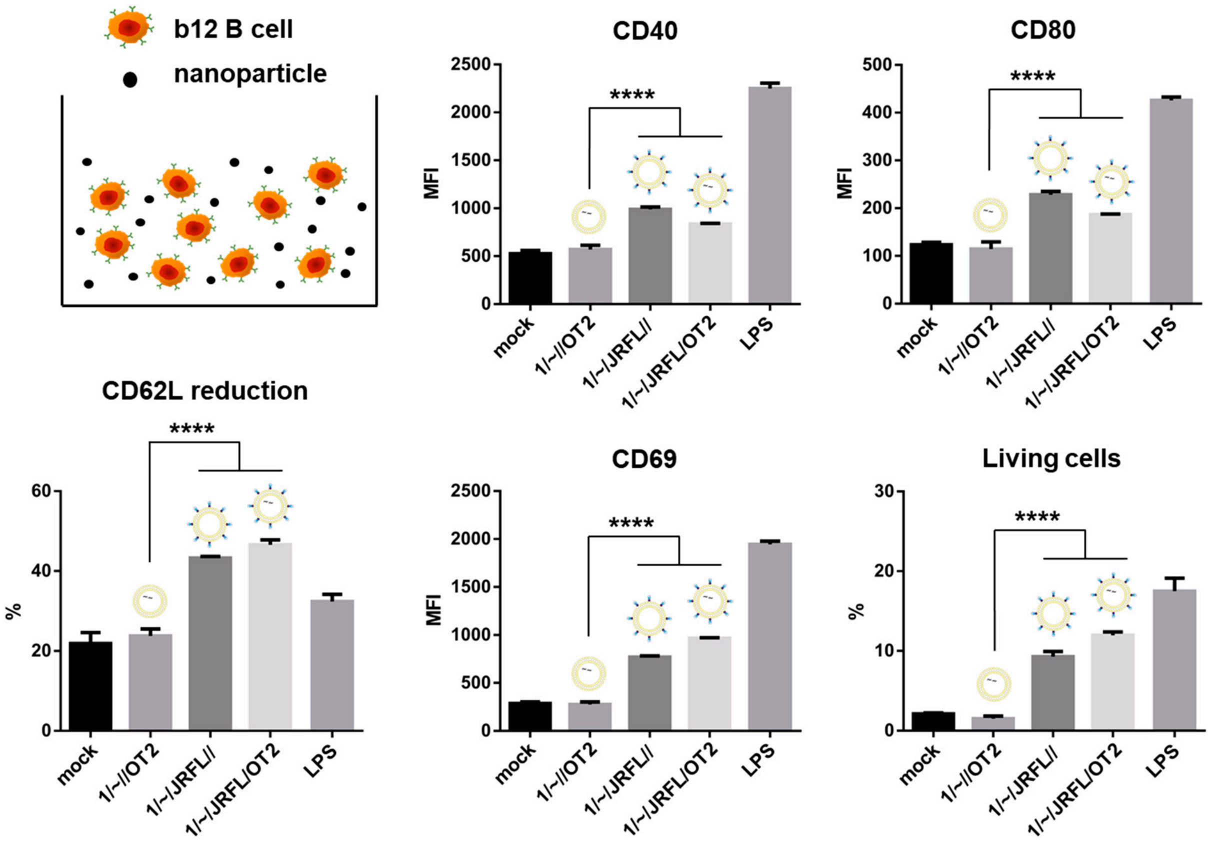

Figure S1. In vitro B cell activation induced by first-generation liposomes

2 x 10^5 Env-specific b12 B cells were incubated for 18 h in the presence of liposomal formulations with a bulk concentration of 1 µg/mL Env. Incubation with LPS was done as a positive control for polyclonal B cell activation. B cell activation was analyzed by FACS based on the upregulation of CD40, CD80 and CD69 as well as the downregulation of CD62 ligand. Additionally, the survival rate of B cells was defined by FACS staining with a viability dye. Error bars indicate means of three independent experiments. **** $p < 0.0001$; ordinary one-way ANOVA with Dunnett's multiple comparisons test.

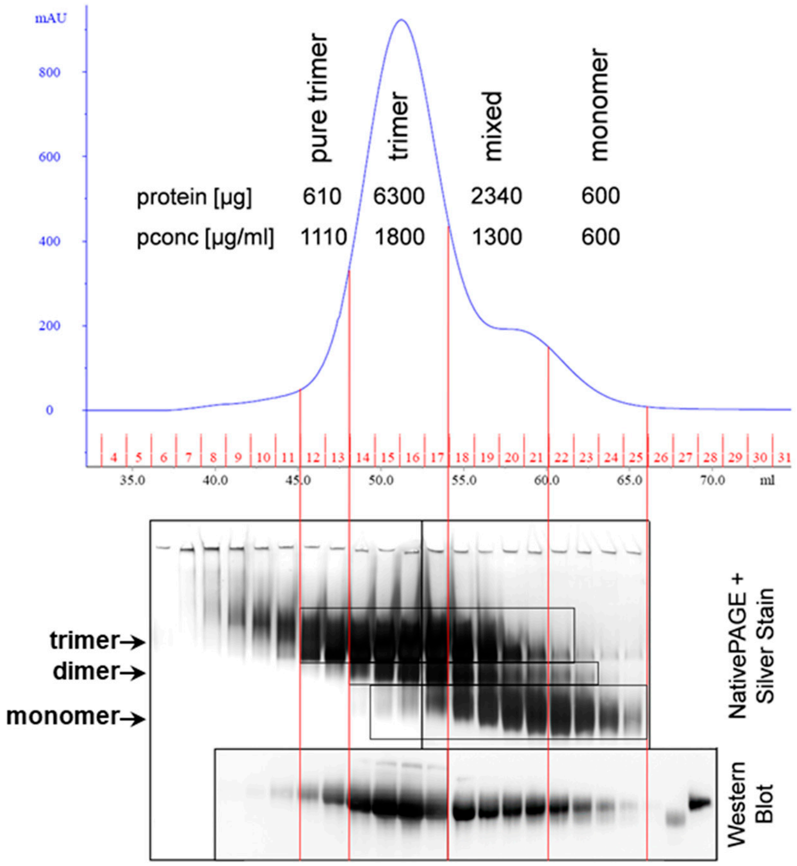

Figure S2. Size-exclusion (SEC) purification of SUFO.664-His.

The trimers were expressed in 293F cells and purified by Lectin affinity chromatography followed by SEC (blue chromatogram). Eluted protein fractions (red) were collected and subsequently analyzed by NativePAGE + silver stain and Western Blot with both gp140 and gp120 reference controls (right). Three boxes indicate the trimer, dimer and monomer lanes on the silver stain image. The chromatogram was divided into four different major fractions resembling the protein purity states and the total amount of protein obtained from 2 liter 293F culture in each major fraction is given in µg.
